# Deciphering pain: molecular mechanisms and neurochemical pathways–challenges and future opportunities

**DOI:** 10.3389/fmolb.2024.1382555

**Published:** 2024-11-14

**Authors:** Bahar Dehghan, Narges Abolhasanzadeh, Behrouz Shademan, Alireza Nourazarian

**Affiliations:** ^1^ Department of Biology, Faculty of Natural Sciences, University of Tabriz, Tabriz, Iran; ^2^ Medical Journalism, School of Paramedical Sciences, Shiraz University of Medical Sciences, Shiraz, Iran; ^3^ Department of Basic Medical Sciences, Khoy University of Medical Sciences, Khoy, Iran

**Keywords:** pain perception, nociceptors, neuroplasticity, signal transduction, precision medicine

## Abstract

This review delves into the intricate biological underpinnings of pain perception. It encompasses nociceptive signaling pathways, the molecular mechanisms involved, and the subjective experience of discomfort in humans. The initial focus is on nociceptor transduction, where specialized neurons transform noxious stimuli into electrical impulses. Subsequently, the review explores the central nervous system, elucidating how these signals are processed and modulated by critical elements such as ion channels, receptors, and neurotransmitters (e.g., substance P, glutamate, GABA). Shifting gears toward chronic pain, the review examines the concept of neuroplasticity, highlighting its potential to induce maladaptive responses through alterations in neural networks. The burgeoning field of pain genomics, alongside established genetic research, offers valuable insights that could pave the way for a framework of personalized pain management strategies. Finally, the review emphasizes the significance of these molecular insights in facilitating accurate therapeutic interventions. The overarching objective is to establish an integrative framework for precision medicine in pain management by incorporating this information alongside biopsychosocial models. This framework serves to translate the heterogeneous landscape of pain mechanisms into a coherent roadmap for the development of effective therapies.

## 1 Introduction

Pain is a complex experience that involves multiple dimensions, including sensory, emotional, cognitive, and behavioral components (Kessler et al., 2020). This review aims to examine the molecular mechanisms and neurochemical pathways underlying pain, with a specific focus on chronic neuropathic pain. We will also discuss the challenges and future opportunities in this field.

Neuropathic pain is a type of chronic pain that occurs due to a lesion or dysfunction in the somatosensory nervous system. According to the International Association for the Study of Pain, neuropathic pain is defined as a condition in which pain arises from damage or dysfunction in the sensory nerve network. This broad definition encompasses more than 100 conditions, covering injuries throughout the entire pain neuroaxis (Kessler et al., 2020). In response to neuropathic pain, the body often tries to protect the affected area until the tissues can heal. However, in cases of chronic neuropathic pain, the brain’s response to the damage is incorrect due to numerous factors involving both the brain and its modulators (Cutts et al., 2021). Consequently, the sensory system becomes imbalanced, leading to misinterpretation of sensory inputs and the spontaneous generation of painful sensations (Obeng et al., 2021). The mechanisms responsible for this type of chronic pain are still not fully understood (Bán et al., 2020).

Diabetic neuropathy, Postherpetic neuralgia (after shingles), Trigeminal neuralgia (facial nerve pain), Radiculopathy (pinched nerve in the spine),Phantom limb pain (after amputation) and Chemotherapy induced neuropathy These conditions can involve damage or dysfunction at various levels of the somatosensory nervous system, from the peripheral nerves to the spinal cord and brain. Pain can be classified as acute or chronic. Acute pain is a normal protective response to actual or potential tissue injury. It is typically short-lived and resolves with healing. In some clinical scenarios, such as after surgery or trauma, acute pain may persist beyond the expected healing period and transition into chronic pain.

Chronic pain, on the other hand, is a persistent pathological condition that no longer serves a biological protective function. It can result from nerve damage or an underlying disease, such as diabetes, cancer, or certain autoimmune disorders. Acute pain is often associated with muscle spasms and stimulation of the sympathetic nervous system. It can be caused by an underlying disease or injury. Although unpleasant, acute pain plays a useful biological role in signaling potential tissue damage and triggering protective reflexes.

Patients suffering from chronic pain may exhibit cognitive deficits, mood alterations, and behavioral changes. Chronic pain is frequently accompanied by psychological states such as anxiety, depression, and sleep disturbances. A key concept in understanding chronic pain is neuronal plasticity. Adaptive changes occur in the nociceptive pathways, leading to peripheral and central sensitization. This sensitization can result in amplification of pain signals, lowered pain thresholds, and exaggerated perception of normally non-painful stimuli (allodynia).

Peripheral sensitization occurs when inflammatory mediators are released at the site of tissue injury, sensitizing the peripheral nerve endings, and increasing their responsiveness to noxious stimuli. Central sensitization involves functional changes in the central nervous system, such as increased excitability of spinal cord neurons and reorganization of cortical maps, contributing to the maintenance and amplification of pain perception.

The innate inhibitory system of nociceptors contains a class of endogenous compounds, such as monoamines, naturally occurring opioids, and cannabinoids (Iolascon and Moretti, 2019). However, the main issue with using opioids for therapy is the potential development of tolerance and physical dependence with prolonged use (Chen et al., 2022). As a result, current efforts in drug development have focused on opioids with fewer side effects. This can be achieved through multifunctional opioids, biased opioid agonism, or allosteric regulation of the opioid receptors (Chen et al., 2022). These efforts include the development of new analgesics that target receptors expressed by adrenergic, cannabinoid, P2X3 and P2X7, NMDA, serotonin, and sigma receptors, as well as ion channels like the voltage-gated sodium channels Nav1.7 and Nav1.8 (Lu and Gao, 2023). Additionally, soluble epoxide hydrolase, sepiapterin reductase, and MAGL/FAAH are enzymes that have been selected as target proteins for the development of novel analgesics (Donnelly et al., 2020).

To summarize, both central and peripheral mechanisms contribute to the development of peripherally induced neuropathic pain (pNP). Recent evidence suggests that the persistence of pNP depends on maladaptive mechanisms in the central nervous system (CNS) (Kessler et al., 2020). In other words, the increased responsiveness afterwards could also be explained by the complex localized process of inflammation that occurs after tissue damage and the activation of nociceptive afferent endings in the tissue. This activation is associated with the release of growth factors, cytokines, prostaglandins, serotonin, and bradykinin. Due to the presence of sensitizing substances in inflamed tissue, there is increased peripheral sensitivity to painful stimulation, known as peripheral sensitization. Many researchers consider this to partly contribute to the increased sensitivity of damaged tissue (primary hyperalgesia) (Chen et al., 2022).

The activation of ion channels in response to specific environmental stimuli allows for the conversion of variable mechanical, thermal, and chemical stimuli into voltage changes in neurons (Yang et al., 2021). Functional differences involve the expression, distribution, and phosphorylation of various forms of ion channels in nociceptive neurons (Huang et al., 2019a). There are also differences in the expression and function of enzymes, receptors, voltage-gated ion channels, and changes at synapses in the nociceptive pathway of the CNS (Deng et al., 2020). Additionally, there are differences in the characteristics of intrinsic membranes and the creation of membrane potential oscillations that cause rhythmic firing even in the absence of a stimulus (Chiang et al., 2020). Neuropeptides play a key modulatory role in the nervous system (Yang et al., 2021). Calcitonin gene-related peptide (CGRP) is one of the important dipeptides in the nociceptive parabrachial afferent pathway, which controls the excitatory drive of CeA neurons (Mercer Lindsay et al., 2021). Neurons within the CeA that produce somatostatin (SOM) and/or corticotropin-releasing factor (CRF) control the principal output functions. However, CRF also operates locally by activating a minority of GABAergic CeA and basolateral amygdala (BLA) neurons (Ma, 2022). Therefore, neuropeptides are useful targets for controlling amygdala activity in pain situations (Russ et al., 2021).

## 2 Pain pathways and molecular mechanisms

### 2.1 Acute and chronic pain

**FIGURE 1:**
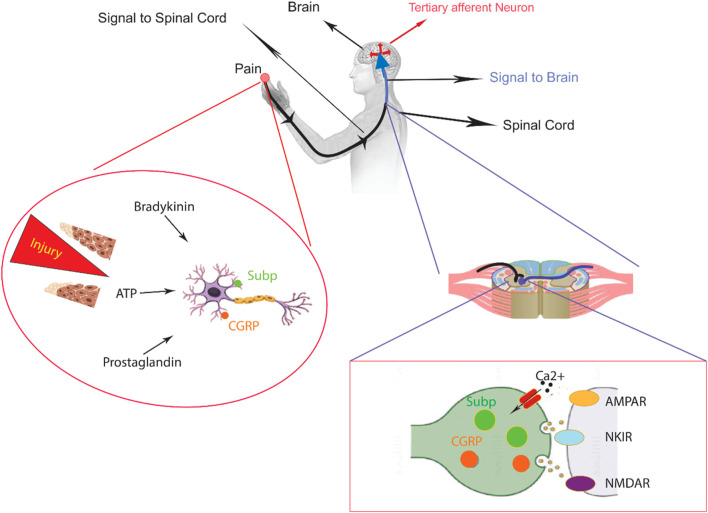
An overview of peripheral nervous system in the sensory pathways leading from the skin to the brain. The skin contains both unmyelinated C-fibers and myelinated Aδ/β-fibers, which are peripheral nerve endings responsible for sensing stimuli. When chemicals like inflammatory mediators and neuropeptides are released from an injury site or the nerve endings themselves, they activate receptors and channels on the neighboring peripheral nerve terminals. This leads to the initiation of an action potential at the initial segment of the axon. The axon of the first-order neuron then enters the spinal cord and contacts the second-order neuron in the gray matter. At this point, an action potential is generated at the initial segment of the second-order neuron and travels along the sensory pathway to specific regions of the brainstem and thalamic nuclei. From the thalamus, the sensory signal reaches the third-order neurons, which then project pain signaling to multiple cortical and subcortical regions.

The nervous system senses thermal, mechanical, and various chemical irritants from either internal or external sources that are largely dichotomous. When intense enough to cause damage, the person experiences pain, which can be either acute or chronic, although both are clinically distinct conditions. Within the context of acute pain, often associated with muscular spasms and sympathetic nervous system stimulation (Figure 1), a clear biological purpose exists. Typically arising from underlying pathology or injury, this transient pain state serves a valuable function.

However, in the chronic pain scenario, the pain transmission pathway traversing both the peripheral and central nervous systems undergoes significant plasticity. This plasticity manifests as sensitization and exaggeration, leading to an amplification of pain signals and heightened sensitivity. While plasticity can be beneficial by facilitating defensive reflexes, its persistence can ultimately culminate in a chronic pain state (Mangnus et al., 2022). Chronic pain, a potential indicator of underlying pathology, is defined as pain persisting beyond the expected healing time associated with injury or illness. Often comorbid with psychological conditions, it lacks a well-defined endpoint and serves no apparent biological purpose.

Peripheral nerve alterations can evoke sustained pain in conditions such as injuries, diabetes, arthritis, and tumor growth. These changes can manifest after nerve fiber damage, leading to potentiated spontaneous firing, altered conduction, and modifications in neurotransmitter properties.

Nociceptors, the body’s primary pain signaling system, transmit signals from the source of injury to the dorsal horn of the spinal cord. Nociceptive pain, exemplified by a pinprick to the foot, activates regions surrounding the nerve terminal. This activation triggers the release of pain-inducing molecules that travel through primary sensory pathways, targeting specific neurons within laminae I, IV, and V of the spinal dorsal horn (Smart et al., 2022). Aβ, Aδ, and C-fibers also reach layers II-VI, but to a much lesser extent, modulating the activity of pain-predicting neurons. Within the context of tissue injury and cutaneous inflammation, neighboring cells situated at the peripheral nerve terminal release inflammatory mediators. It is paramount to distinguish this from the subjective experience of pain perception itself. These mediators, including adenosine and its phosphorylated derivatives (AMP, ADP, and ATP), bradykinin, glutamate, histamine, interleukin 1 and 6, serotonin, platelet-activating factor, nerve growth factor, prostaglandin E2, and tumor necrosis factor-α, serve to sensitize nociceptors, thereby amplifying pain perception.

Nociceptive signals, originating from the dorsal root ganglia (DRG), are relayed to the dorsal spinal cord, brainstem, and the brain, contributing to the conscious experience of pain (Cutts et al., 2021).

The DRG is made up of pseudounipolar sensory neurons. Each of these neurons has a single axon that splits into two separate branches. One branch connects with peripheral tissues, while the other branch projects centrally and ends in the dorsal horn of the spinal cord. It is important to note that nociceptive neurons in the DRG can be divided into two main types based on the size of their cell bodies: large-diameter and small-diameter neurons. Studies using immunohistochemical staining have shown that there is a correlation between cell body size and fiber type. Nociceptive neurons that transmit signals through C and Aδ fibers have small cell bodies, whereas those that use Aβ fibers tend to have larger cell bodies (Obeng et al., 2021).

### 2.2 Nociception

Nociceptors, widely distributed throughout the body, serve as the primary sensory transducers of pain. These specialized receptors are present in the skin, musculoskeletal structures (periosteum, joint capsule, ligaments, and muscles), the cornea of the eye, and dental pulp. Additionally, they are abundantly found in internal viscera, including the meninges, pleura, peritoneum, and organ walls. Nociceptors function by transmitting nociceptive information to the brain in response to a variety of stimuli, encompassing biological, electrical, thermal, mechanical, and chemical agents. This integrated sensory processing culminates in the perception of pain (Bán et al., 2020). Pain is initially perceived in the thalamus and then transmitted to the limbic system and cerebral cortex for interpretation. Nociceptors, located at the end of nerve fibers, are responsible for pain transmission. Two types of fibers, Aδ and C, participate in this process. Aδ fibers primarily produce sharp, well-defined pain, which is typically experienced in response to physical traumas such as cuts, electrical shocks, or blows. The nerve fibers discussed in this passage can be classified into two types: myelinated and unmyelinated. Myelinated fibers can transmit an action potential at a speed of about 20 meters/second to the CNS. These fibers do not have opioid receptors, but constant vigilance is maintained over pain receptors located at the ends. Unmyelinated C-fibers, on the other hand, are very thin and vulnerable to damage. They conduct painful stimuli very slowly, at 0.5–2 m/s. Many C-fibers combine to form a “net” (Kessler et al., 2020). C-fibers exhibit a broad and poorly defined distribution within the body. These unmyelinated nociceptors respond to a diverse range of stimuli, including mechanical forces, thermal extremes, and chemical agents. Patients experiencing C-fiber-mediated pain often describe it as sharp, fleeting, and pulsating. Notably, the distal terminals of C-fibers express various receptor proteins, most importantly opioid receptors. These receptors are synthesized within the DRG cell bodies and trafficked along the axons to the synapses located at the spinal cord dorsal horn and peripheral tissue nerve endings. It is important to acknowledge that these receptors exist in a dormant state within the cell membrane of nerve endings, often referred to as “silent” receptors (Iolascon and Moretti, 2019). The inflammatory process can trigger the activation of previously dormant opioid receptors on C-fibers. Cytokines released by inflammatory cells have the capacity to breach the compromised perineurium and stimulate these receptors. Consequently, both endogenous and exogenous opioids can bind to and sensitize the activated opioid receptors. Furthermore, prostaglandins can independently contribute to C-fiber sensitization. It is noteworthy that nonsteroidal anti-inflammatory drugs (NSAIDs) exert their analgesic effects by reducing prostaglandin synthesis, thereby elevating the pain threshold, and diminishing C-fiber sensitivity. Corticosteroids, on the other hand, target the inflammatory process itself, reflecting a fundamental defense mechanism mediated by crosstalk between the immune and nervous systems.

Pain perception typically unfolds in two distinct phases. The initial phase is characterized by the rapid transmission of signals via Aδ fibers, followed by a secondary phase mediated by C-fibers. Notably, pain serves as a crucial warning signal, alerting the organism to potential tissue damage. It is, therefore, an essential physiological process (Iolascon and Moretti, 2019). In certain chronic pain presentations, the etiology may not be attributable to nociceptor activity or a clear peripheral or CNS pathology. This type of clinical pain deviates significantly from physiological pain in both its character and manifestation. Functional magnetic resonance imaging (fMRI) studies have revealed altered activity patterns within brain regions associated with mood, affect, and behavioral regulation in chronic pain patients. It is hypothesized that individuals experiencing significant psychosocial stressors, such as those associated with independent living away from home, may exhibit a heightened susceptibility to developing chronic pain (Chen et al., 2022). Pain perception is a complex process orchestrated by a diverse array of receptors functioning at various stages of the nociceptive pathway. Targeting these receptors during pain transduction holds promise for the development of novel analgesic strategies. The specific anatomical location of pain following sensitization influences the repertoire of receptors activated. This includes transient receptor potential (TRP) ion channels (TRPV1, TRPV2, TRPM8), Piezo type 2 channels, acid-sensing ion channels (ASICs), purinergic receptors (P2X and P2Y), bradykinin receptors (B1 and B2), AMPA and NMDA glutamate receptors, metabotropic glutamate receptors (mGluRs), neurokinin 1 (NK1) receptors, and CGRP receptors (Chen et al., 2022). While the simultaneous blockade of TRPV1, TRPV2, TRPM8, Piezo type 2, ASICs, P2X, P2Y, bradykinin B1 and B2 receptors, AMPA and NMDA glutamate receptors, metabotropic glutamate receptors, neurokinin 1 receptors, and CGRP receptors demonstrates therapeutic value in experimental pain models, it may not be a clinically viable approach due to potential side effects. Conversely, other studies have shown promise by activating opioid, adrenergic, serotonergic, and cannabinoid receptors. These receptors mediate local inhibitory control, thereby modulating both central and peripheral processing of painful stimuli. However, achieving long-term suppression of the transition from acute to chronic pain remains a significant challenge (Lu and Gao, 2023).

### 2.3 The role of ion channels, neuropeptides, and neurotransmitters in pain transmission

**FIGURE 2:**
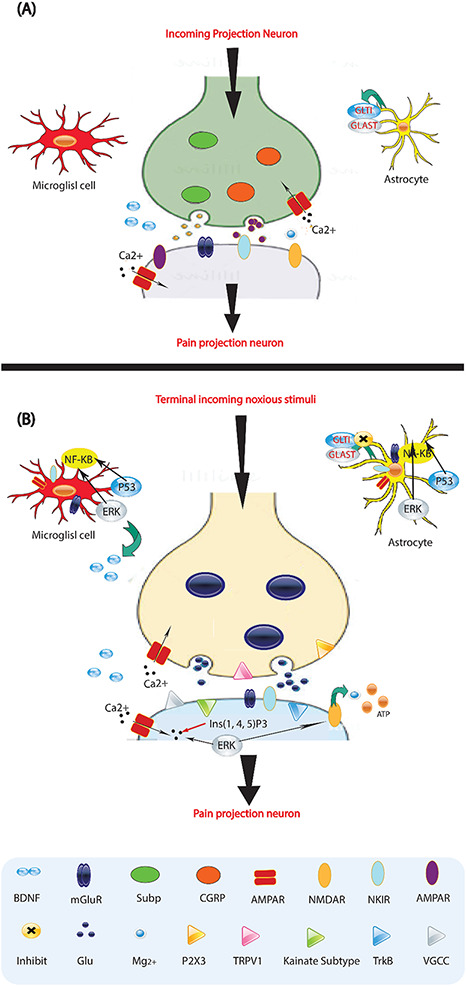
Molecules involved in pain processing. **(A)** Low-frequency activation of Ad and C-fiber nociceptors by mild noxious stimuli leads to the release of glutamate (Glu) from the central presynaptic afferent nerve terminals in the spinal cord dorsal horn. This results in the short-term activation of AMPA (α-amino-3-hydroxy-5-methyl-4-isoxazole proprionic acid) and kainate subtypes of ionotropic glutamate receptors. Although the NMDA (N-methyl-D-aspartate) ionotropic glutamate receptor subtype (NMDAR) is also present, it remains silent due to being blocked by Mg2+. This signaling to dorsal horn pain-projection neurons provides information about the onset time, duration, and intensity of noxious stimuli from the periphery. Both astrocytes and microglia are unaffected by these synaptic events. **(B)** After repetitive synaptic communication, which can occur following a short barrage of nociceptive afferent input, there is an increase in the responsiveness of dorsal horn pain-projection neurons to subsequent stimuli, known as central sensitization. This is mediated by the co-release of glutamate and neurotransmitters such as substance P (sub P) and CGRP, which activate NMDAR and result in voltage-gated Ca2+ currents (VGCCs). Additionally, inositol-1,4,5-trisphosphate (Ins (1,4,5) P3) signaling and mitogen activated protein kinases, such as extracellular signal-regulated kinase (ERK), p38, and c-Jun N-terminal kinase (JNK), are activated. In neurons, ERK can further sensitize excited AMPA receptors (AMPARs) and NMDARs. Activation of purinoreceptors (P2X3) by ATP, activation of substance P receptors (the neurokinin 1 receptor (NK 1R)), activation of metabotropic glutamate receptors (mGluR), and release of brain-derived neurotrophic factor (BDNF) all contribute to enhanced nociceptive transmission. Astrocytes and microglia express various neurotransmitter receptors and are activated by glutamate, ATP, and substance P. At synapses, the glutamate transporters, glutamate transporter 1 (GLT1) and glutamate aspartate transporter (GLAST), which are crucial for clearing synaptic glutamate, become dysregulated after prolonged exposure to elevated levels of synaptic glutamate. Ongoing excitation can induce ERK, p38, and JNK activation in microglia and astrocytes. Each of these kinases can activate the transcription factor nuclear factor-κB (NF-κB), which induces the synthesis of inflammatory factors. Upregulation of the V1 transient receptor potential channel (TRPV1) after inflammation further contributes to the sensitization to noxious signals. During this time, normally non-nociceptive Ab fibers can also activate pain-projection neurons.

Persistent pain after a nerve injury occurs because the properties of a peripheral nerve change. The sensory neurons translate mechanical, thermal, and chemical stimulation into voltage changes using ion channels that detect specific stimuli from the environment (Figure 2). Additionally, various ion channels in peripheral nerves, DRG, and receptors, enzymes, and voltage-gated channels are also expressed, distributed, and phosphorylated, leading to changes associated with neuropathic pain (Donnelly et al., 2020). These changes affect synapses in the nociceptive pathway of the CNS, resulting in variations in intrinsic membrane properties and the generation of membrane potential oscillations. These oscillations lead to rhythmic firing, even without a stimulus. Recent studies have found that N- and L-type calcium channels participate in the release of CGRP from injured nerve terminals *in vitro*. Blocking N-, T-, and P-type calcium channels has shown to block all types of experimental neuropathic pain. This suggests that calcium channels play a role in the expression of the neuropathic state (Kessler et al., 2020). Selective blockers of calcium channels such as gabapentin, oxcarbazepine, lamotrigine, and ethosuximide may be highly effective in treating neuropathic pain. Importantly, Cummins et al. demonstrated increased density and upregulation of membrane sodium channels in injured DRG axons near the nerve transection site. Six subtypes of sodium channels have been identified in the DRG, and blocking these subtypes may be crucial in pharmacotherapeutics for patients with neuropathic pain (Donnelly et al., 2020).

Narcotics, also known as opioids, indeed play a significant role in modulating various functions within the nervous system, both directly and through interactions with other neuropeptide and neurotransmitter systems. This modulatory effect contributes to their potent analgesic properties as well as their potential for abuse and addiction (Chen et al., 2022).

The density of these substances, which are neuropeptides in the brain, is highest among the limbic areas that participate in learning, memory, and emotion. In this review, we will focus on the amygdala, an important limbic region that plays a key role in emotional-affective behavior and pain modulation. The dendritic circuitry of the amygdala, particularly, has been extensively studied. The amygdala contains several nuclei and structures, such as the basolateral amygdala (BLA) and central (CeA) nuclei, as well as intercalated cells (ITC) between them, that have been implicated in pain-related functions. While many neuropeptides are expressed throughout the amygdala, we will only discuss the neuropeptides that have gained clinical interest as mediators of nociceptive processing, specifically those present in sizable amounts within the CeA. One important peptide is the CGRP, which is located on the excitatory drive of CeA neurons from the afferent nociceptive pathway originating in the parabrachial area. CRF and/or somatostatin (SOM) are also expressed by this nucleus, and they act locally to excite neurons in the CeA and BLA as long-range projections, providing an important output function. For example, the neuropeptide peptide S (NPS) is present in inhibitory ITC neurons that regulate amygdala output. Oxytocin and vasopressin modulate the amygdala output in opposite directions (Chiang et al., 2020). Amygdala function is influenced by endogenous neuropeptides via the opioid system of mu, delta, and kappa receptors (MOR, DOR, and KOR) and their peptide ligands (β-endorphin, enkephalin, and dynorphin), which can have complex and sometimes opposing effects. The amygdala, which is rich in neuropeptides, is associated with the emotional aspects of pain and pain regulation. Neuropeptides can directly (CGRP-R, CRF1-R, MOR, DOR, V1aR) or indirectly (via excitatory (MOR, DOR) and inhibitory (CGRP-R, CRF1-R, NPS-R, OT-R) synaptic drives) regulate amygdala output neurons, making them an interesting target (Sun et al., 2020). Peptide systems, in general, aside from the presumed OT-R, do not have significant involvement, leaving open the possibility that they could be viable targets in pain and other conditions, despite the release requirements associated with neuropeptides. In conclusion, the evidence strongly suggests that NPS-R activation, but not CGRP-R, MOR, CRF1-R, KOR, and V1a-R activation, may have beneficial effects in pain conditions. Another notable forebrain structure involved in learning and memory functions is the anterior cingulate cortex (ACC), which has receptors present in this part of the brain (Barik et al., 2021). Recent research also indicates that the prefrontal cortex is activated by painful stimuli, and brain chemistry in that region becomes altered in patients with chronic pain (Sugimura et al., 2016).

CNS components involved in pain transmission and modulation, from the spinal cord to the ACC, have been shown to be highly plastic. These modifications occur rapidly and are long-lasting after injury. Patients with chronic pain often report deficits in memory and concentration. However, the synaptic circuitry underlying these experiences is undefined. N-acetylaspartylglutamate (NAAG) is one of the most abundant and widely distributed peptide neurotransmitters in the mammalian nervous system (Sugimura et al., 2016). At presynaptic sites, NAAG binds to the metabotropic glutamate mGlu3 receptor. This binding inhibits the release of glutamate and other neurotransmitters. NAAG also binds to mGlu3 receptors on glial cells, stimulating the release of neuroprotective growth factors from these cells. It is important to note that glutamate release from neurons is associated with various pathologies, including stroke, traumatic nervous system injury, amyotrophic lateral sclerosis, inflammatory and neuropathic pain, diabetic neuropathy, and schizophrenia-like symptoms induced by phencyclidine (Yang et al., 2021). NAAG is inactivated after synaptic release by behaviorally specific peptidases. Novel compounds that selectively inhibit these enzymes would prolong the activity of synaptically released NAAG. These compounds have shown significant therapeutic efficacy in animal models for various clinical conditions. The selective peptide neurotransmitter mGlu3, derived from the NAAG receptor, enhances the inhibition of glutamate release from neurons and astrocytes stimulated by TGF-β (Huang et al., 2019b). Animal modeling results to date indicate that inhibiting GCPII and GCPIII, which increases levels of endogenously released NAA, exhibits therapeutic efficacy in a range of important human nervous system disorders. Future research in this therapeutic direction should focus on the further development of novel NAAG peptidase inhibitors that can permeate the blood-brain barrier. Additionally, further exploration should be conducted to understand the functional role of NAAG itself at postsynaptic mGlu3 receptors, as well as the significance of glial cells in mGlu3 receptor-mediated NAAG responses (Deng et al., 2020).

## 3 Neurochemicals and pain control

### 3.1 Gate control theory of pain

In 1965, Melzack and Wall’s groundbreaking work introduced a new framework for understanding pain perception. They questioned the existing linear model, which suggested a direct pathway from peripheral stimulation to central pain processing. Instead, they proposed a “gate control” mechanism situated within the dorsal horn of the spinal cord. This mechanism regulates incoming nociceptive signals, thus controlling the intensity of pain experienced in the higher brain centers. Melzack and Wall’s work was particularly significant because it emphasized how emotions, cognitive state, and past experiences influence this gating process. By linking physiology with psychology, their theory revolutionized pain research and shed light on the intricate interplay between sensory and psychological factors in pain perception. Moreover, this groundbreaking work laid the foundation for the concept of pain modulation, which is now a cornerstone of contemporary pain management strategies (Deng et al., 2020; Puopolo, 2019; Li C. et al., 2019).

The gate control theory of pain suggests that there is a modulatory mechanism in the spinal cord that regulates the transmission of nociceptive signals. This mechanism is often referred to metaphorically as a “gate,” and it is influenced by both descending pathways from the brain and ascending inputs from the site of tissue damage or inflammation. According to this theory, the perception of pain is not solely determined by the intensity of the noxious stimulus but can be influenced by numerous factors. These factors include attention, emotional state, and cognitive processes, which could either amplify or inhibit the transmission of pain signals along the ascending pathway [43.44].

Despite their established role in pain management, both opioids and cannabinoids present ethical considerations. Opioids pose a significant risk of dependence, overdose, and misuse, contributing to the current public health crisis. Careful prescribing practices and diligent monitoring are crucial to mitigate these risks. Similarly, while cannabinoids demonstrate potential analgesic properties, their legal ambiguity and potential for abuse raise ethical concerns regarding their use in clinical settings. The responsible prescription of these substances, coupled with informed consent from patients and strict adherence to regulatory guidelines, is paramount for their ethical implementation in pain management.

### 3.2 Endogenous pain control systems

The hypothalamus, amygdala, and ACC are the main neuroanatomical circuits involved in the regulation of pain. These circuits project to brainstem nuclei such as the rostral ventral medulla and locus coeruleus, as well as the midbrain periaqueductal gray (Li C. et al., 2019). The transmission of sensory neurons can be altered by internal modulatory mechanisms. Activation of α2-adrenergic, opioid (μ), and cannabinoid (CB1) receptors can modulate the signaling of acute and chronic pain. This is achieved through various monoaminergic transmitters, including noradrenaline (NA), dopamine (DA), serotonin, endogenous opioids (met- and leu-enkephalins and dynorphins), and cannabinoids (anandamide). These substances are part of the innate pain relief system (Ziółkowska, 2021).

#### 3.2.1 Amphetamines promote the release of endogenous monoamines (noradrenaline)

The sympathetic nervous system is constantly active throughout the body. It affects the release of NA and acts on presynaptic modulation. The activity of pain modulation depends on the specific organ being considered. Spinal NA primarily comes from noradrenergic fibers in areas A5, A6, and A7. The dorsal horn of the spinal cord is where nociceptive information is integrated and modulated. Along noradrenergic fibers in the spinal cord, there are varicosities that do not form synapses. This maintains a nonsynaptic condition where terminal axons connect with target cells, such as sensory neurons expressing α2-adrenoceptors. When norepinephrine is released from synaptic and nonsynaptic boutons, it diffuses widely and can completely block the release of glutamate from spinal nociceptive primary afferent fibers. α2-adrenoceptor antagonists can counteract this action. Therefore, the release of norepinephrine into the extracellular space may assist in pain processing (Yang et al., 2021).

Norepinephrine from nerve terminals inhibits other transmitters presynaptically. NA release increases during ischemia and is regulated by α2A receptors through negative feedback. In DRG neurons, it has been shown that NA strongly sustains the activity of TRPV1 channels (capsaicin-sensitive ion channels). Yohimbine, a selective α2 antagonist, can replicate and subsequently counteract this effect, suggesting the involvement of α2 adrenergic receptors (Huang et al., 2019c).

There is compelling evidence that both in human spinal and epidural anesthetic models and animal models of neuropathic pain, the administration of α2-adrenoceptor agonists (clonidine and dexmedetomidine) in the spinal cord leads to analgesia. A meta-analysis demonstrated that intraoperative dexmedetomidine administration reduces postoperative pain perception (Tavakolian-Ardakani et al., 2019).

Amphetamines cause noradrenergic and dopaminergic neurons in the frontal cortex and nucleus accumbens to release NA and DA from their reserves. Amphetamines are also known as analgesics, so it is reasonable to assume that their effects on pain management may be related to the release of DA and NA. D2-like receptors in the limbic system (nucleus accumbens) improve analgesic efficacy. In a rat model of neuropathic pain, ascending activation of dopamine (DA) release from DA neurons to the mesolimbic A11 dopamine cell group of the spinal cord dorsal horn inhibits allodynia via stimulation of α2-adrenoceptors and D2 receptors (Azzouz et al., 2019).

Moreover, neuropathic pain has been effectively treated with antidepressants such as tricyclic antidepressants and serotonin/noradrenaline reuptake inhibitors. These findings support animal studies suggesting that noradrenergic and dopaminergic mechanisms underlie the anti-allodynic effect in neuropathic pain. Selective serotonin reuptake inhibitors (SSRIs) do not effectively treat chronic pain; however, fluoxetine, a selective serotonin reuptake inhibitor, is widely used for nociceptive pain, inflammatory pain, and opioid tolerance and dependency. The action of fluoxetine may be attributed to its antagonistic activity on nonsynaptic NR2B receptors, leading to neuroprotective effects (Durairaj et al., 2018).

#### 3.2.2 Endogenous opioids include met- and leu-enkephalins, β-endorphin, and dynorphins

Derivatives of the opium poppy have been used for their analgesic effects for many years. This use was first described and publicized in the Homeric Iliad and Odyssey. The existence of two brain pentapeptides (met- and leu-enkephalins) capable of binding morphine receptors was later discovered, followed by the discovery of β-endorphin and dynorphin. These discoveries further elucidated their functions in the innate pain system and immune signaling (Niyonambaza et al., 2019). Endogenous opioids, with the aid of μ-, δ-, and κ-receptors, serve as a modulating factor in the innate analgesic system. They affect various pathways in the brain, including the reward pathway in the limbic system, decision-making pathways, and inhibitory control in lateral and ventrolateral medullary regions, which can cause respiratory depression. They also inhibit acetylcholine release from Auerbach’s plexus in the gut, leading to constipation, and affect the release of other transmitters via naloxone-sensitive receptors. Endogenous dynorphin at the amygdala opioid receptor (KOR) has been implicated in mediating chronic pain and inducing stress effects (Mobed et al., 2020). Similarly, blocking opioid receptors (KOR) may help prevent anxiety associated with surgery and its outcomes, such as increased stress before the procedure.

#### 3.2.3 Endogenous cannabinoids

Patients with chronic pain have been using Cannabis as a medicinal plant for thousands of years. Cannabis is an illegal drug derived from the marijuana plant or Cannabis sativa. The discovery of Δ9-tetrahydrocannabinol (THC) as the main psychoactive compound in marijuana led to the development of synthetic cannabinoids and the identification of their receptors: CB1 and CB2 (Banerjee et al., 2020). THC, along with other Cannabis chemicals and endogenous/exogenous cannabinoids, has been used to treat pain. The endocannabinoid system plays a role in modulating pain in the CNS. Anandamide and 2-arachidonoylglycerol (2-AG) are two endogenous neuromodulators for CB1 receptors in neuropathic pain. CB1 and CB2 receptors, which are G-protein coupled receptors, regulate mood, hunger, memory, perception of pain, and immune system function (Da et al., 2022). CB1 receptors are present in various organs, including the cerebellum, basal ganglia, cerebral cortex, and nucleus accumbens. CB2 receptors have higher expression levels in microglia and immune cells and their activation has been associated with anti-inflammatory effects, such as reduced neutrophil and macrophage counts and decreased inflammatory cytokine output. CB1 receptors are expressed primarily on glutamatergic and GABAergic axon terminals, and cannabinoids have been shown to inhibit GABA release from interneurons expressing CCK and Glu release from cerebellar parallel fibers in CCHR (Chauhan et al., 2020). These findings demonstrate how peptides function at presynaptic locations via Cav2.2 N-type channels, preventing Ca2+ from entering the axon terminals. Activation of TRPV1 receptors leads to tonic depolarization of hippocampal interneurons, resulting in the synthesis and release of endocannabinoids that mediate retrograde depression of glutamate release from the same or neighboring presynaptic terminals. This example illustrates analog and nonsynaptic modulation of neurochemical interactions across the brain, independent of discrete pre- and/or postsynaptic activity, in this case through retrograde signaling from the postsynaptic site releasing cannabinoids (2-arachidonoyl-glycerol) to activate presynaptic CB1 receptors localized on nonsynaptic nerve terminals. Cannabinoids have been observed to inhibit GABA release, a neurotransmitter that can decrease dopamine release in the nucleus accumbens. When CB1 is triggered, the inhibition is relaxed, resulting in an excess of dopamine release. Cannabis achieves this action by expressing presynaptic CB1 receptors on GABAergic interneurons. Cannabis is used as an alternative to opioids for the management of chronic pain, and places in the United States where medicinal Cannabis usage is permitted have lower death rates from opioid overdoses (Su et al., 2020). ATP is an extracellular signaling molecule, along with other nucleotides and nucleosides. Plasma membrane P2X and P2Y receptors have ionotropic and metabotropic activities mediated by extracellular ATP. Presynaptically, theophylline-inhibitable adenosine, AMP, ADP, and ATP reduce transmitter release in a theophylline-inhibitable manner. These receptors are purinergic receptors of subtype A1. The involvement of P2 and adenosine receptors in pain conduction has been demonstrated, as well as the designation of P2X3 as a sensory transducer. It is worth noting that P2X receptors may be linked to the analgesic effects of acupuncture. Recent studies on drugs acting on P2 receptors have shown the potential for creating highly effective analgesics for peripheral neuropathy (Ou et al., 2019; Madhurantakam et al., 2020).

## 4 Inflammation and neuropathic pain

**FIGURE 3:**
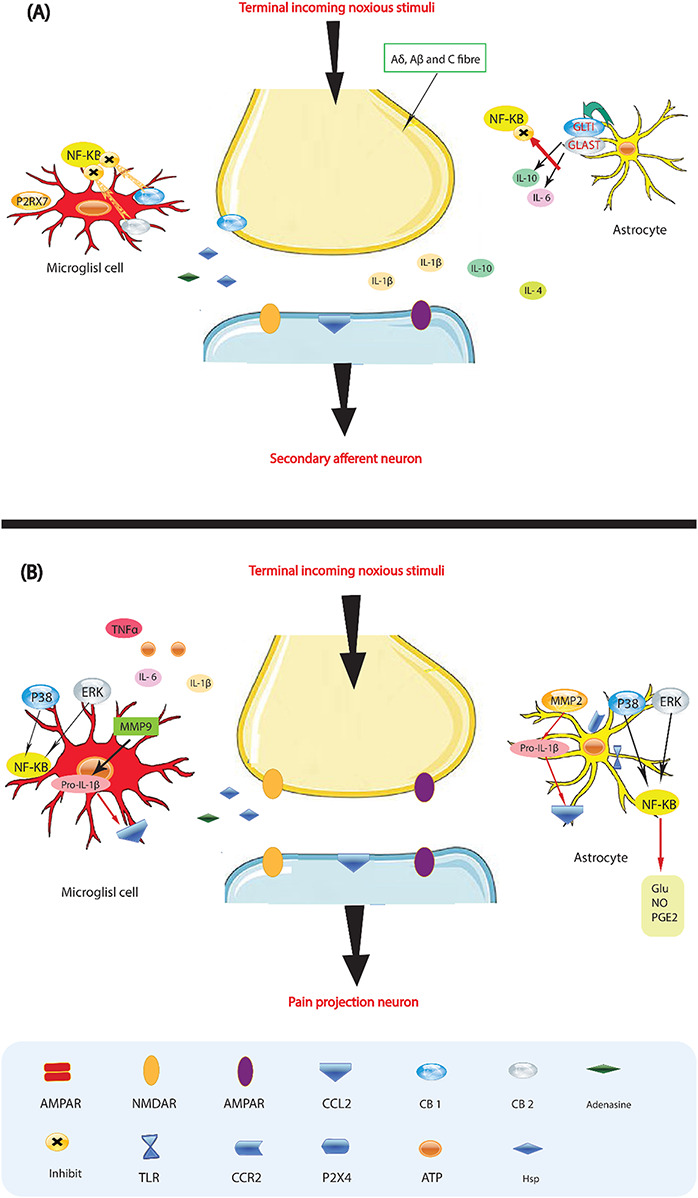
Glia activation: from pro-inflammatory to anti-inflammatory states. **(A)** Pro-inflammatory roles for glia. If a noxious input persists, such as during chronic inflammation or nerve damage, sustained central sensitization leads to transcriptional changes in dorsal horn neurons that alter these neurons’ function for prolonged periods. Astrocytes respond to this ongoing synaptic activity by mobilizing internal Ca^2+^, leading to the release of glutamate (Glu), ATP that binds to P2X 4, tumor necrosis factor-α (TNFα), interleukin 1b (IL-1b), IL-6, nitric oxide (NO) and prostaglandin E2 (PGE2). Activated microglia are also a source of all these pro-inflammatory factors. Matrix metalloproteinase 9 (MMP9) induces pro-IL-1b cleavage and microglial activation, whereas MMP2 induces pro-IL-1b cleavage and maintains astrocyte activation. The activation of p38 mitogen activated protein kinase (p38 MAPK) is induced in both microglia and astrocytes on IL-1b signaling. Astrocytes and microglia express the chemokine receptors CX3CR1 (not shown) and CCR2 and become activated when the respective chemokines bind. After nerve damage, heat shock proteins (HSPs) are released and can bind to Toll-like receptors (TLRs) expressed on both astrocytes and microglia, leading to the further activation of these cell types. **(B)** Activated glia can also be neuroprotective, as they release anti-inflammatory cytokines such as IL-10 and IL-4 and express cannabinoid receptors (CB1 and CB2) that have been shown to exert anti-inflammatory functions and to inhibit microglial toxicity by suppressing chemotactic responses and MAPK signal transduction, with consequent pro-inflammatory cytokine inhibition. The glutamate transporters, glutamate transporter 1 (GLT1) and glutamate–aspartate transporter (GLAST), can resume normal glutamate clearance. Activation of microglial P2RX7 purinoreceptors by ATP leads to TNFα release that protects neurons from glutamate-induced toxicity. Activated astrocytes reduce the spread of tissue degeneration after direct injury through the controlled removal of dying neurons and tissue debris, another neuroprotective effect. In quiescent cells (not shown), nuclear factor-κB (NF-κB) is sequestered in the cytosol by inhibitor of κb (IκB), which binds to specific regions on NF-κB and thereby prevents exposure of the nuclear-localization signal. On stimulation with pro-inflammatory cytokines, IκB proteins are phosphorylated, leading to their ubiquitindependent degradation. Therefore, NF-κB translocates to the nucleus and binds to elements in the promoters of target genes, leading to activation of pro-inflammatory cytokine genes.

According to recent studies, there may be an interaction between the immune system and the neurological system. It has been suggested that inflammation at the site of injured or damaged nerves may be the underlying cause for various forms of neuropathic pain (Xu et al., 2020). When tissue damage occurs, a chain reaction is triggered, leading to the accumulation and activation of innate immune cells (Figure 3). This results in a widespread immune response, as well as localized effects and the production of immunoreactive chemicals such as chemokines, neurotrophic factors, and cytokines (Tilley et al., 2021). Glial cells in the brain and spinal cord, which play a role in nociception, may react to the subsequent neuroinflammatory environment (Bohren et al., 2022). Glial cells, also known as neuroglia, are non-conducting cells that affect the function of synaptic neurons. When microglial cells are activated by peripheral nociceptive signals from nerve damage, pro-inflammatory cytokines such as tumor necrosis factor-a, interleukin-1b, and interleukin-6 are released, influencing the onset of pain (Minhas and Iqbal, 2019). This neuroinflammation then spreads as microglia recruit more cells, which in turn activate nearby astrocytes, leading to an inflammatory condition that culminates in neuropathic pain (Donnelly et al., 2020). Various mediators in this pathway of neuropathic pain have been identified. It has been observed that neurotrophic factors and cytokines can directly affect neurons or stimulate glial cells, thereby influencing pain (Pöyhönen et al., 2019). However, there is conflicting data on the impact of these mediators, despite the efforts of several researchers to understand their role in pain (Chang et al., 2019). Interestingly, it is sometimes possible for one drug to block a pain pathway while another may promote it. Therefore, more extensive research is needed to understand the specific changes in gene expression that contribute to chronic neuropathic pain, as well as the pathways involved. Additionally, increased electrical afferent input to the dorsal horn, including increased discharge from C-fibers, is an important aspect of this neuroinflammatory response, further enhancing the process of central sensitization (Whone et al., 2019). As a result, the conventional approach to pain management has focused on using medication to modify the release of chemical neurotransmitters. As mentioned earlier, the nervous system relies on both chemical and electrical pathways for signaling (Rosich et al., 2017).

Consequently, attenuating comparable electrical impulses using external electrical signals may have a therapeutic effect. It has been established that the immune system and glial cells may play a role in the initiation and maintenance of chronic neuropathic pain (Gui et al., 2017). Indeed, the impact of glial cell activation on the neuroinflammatory process seems to be significant. Therefore, therapeutics that reduce the rate of nociceptive behaviors by manipulating these specific cells will remain among the most promising areas for further research. There are several ways to stimulate glial cells, including (Walker and Xu, 2018) (A). Ravikumar et al. (2015) reported that ATP released by afferent neurons causes migration and activation of microglia within a range of 50–100 mm. This leads to an intracellular increase in Ca2+ and BDNF, resulting in the activation and translocation of NF-kB to the nucleus. These processes initiate the expression of numerous pro-inflammatory agents (Walker and Xu, 2018; Ayanlaja et al., 2018). The subsequent activation of glial cells within the CNS occurs due to the NO-stimulated production of substance P by sensory neurons exposed to painful stimuli or following stimulation of neurokinin-1 (NK-1) receptors with substance P. Other chemical mediators that may induce glial cell activation include direct interaction of purinergic agents, glutamate, and opioid peptides with certain membrane receptors (Ayanlaja et al., 2018).

(B). Intracellular and extracellular ion concentrations can affect glial activation. Increased input from neurons, leading to elevated extracellular K+ levels, causes astrocytes to accept K+ through enhanced mechanisms. This uptake results in membrane depolarization, morphological changes, and potential activation of the astrocytes. The activation of neighboring glial cells may also be influenced by pro-inflammatory factors produced and secreted by the activated glial cell (Stanga et al., 2020). Neuropathic pain can be managed by astrocytes that are activated supraspinally and further activated with glial cells in the spinal cord via descending pathways (Chen et al., 2000). Glial cells do not mediate acute pain caused by injuries like paper cuts or needle stings. However, in more severe injuries, glial cells exhibit dynamic plasticity, transitioning from dormant to active cells with the ability to regulate neuronal activity. During activation, glia undergoes morphological changes including hypertrophy and potential retraction of processes. They also produce specific cell markers and kinases, some of which directly contribute to the initiation and intensification of an immune response (Ortmann and Hellenbrand, 2018).

## 5 Therapeutic strategies for pain management

The management of pain is a crucial aspect of symptom management in various diseases across all medical fields. Pain management can be achieved through pharmacological or non-pharmacological means.

### 5.1 Pharmacological treatments for neuropathic pain

The World Health Organization (WHO) advocates for a multi-step approach to pain management, known as the analgesic ladder (Zobdeh et al., 2022). This strategy prioritizes initiating treatment with medications that are less potent and have a lower risk of side effects. If these initial medications prove inadequate, the approach allows for gradual escalation to more powerful drugs with potentially more severe harmful effects. Importantly, the analgesic ladder encompasses not only pharmacological interventions but also non-pharmacological methods like surgery and physiotherapy (Hou et al., 2021). The first tier of the pharmacological approach typically involves medications like acetaminophen or aspirin, assuming normal liver function (Moisset et al., 2020). For pain associated with inflammation or secondary bone metastases, NSAIDs are often employed (Moisset et al., 2020). The selection between different NSAIDs considers factors like anticipated benefits, potential side effects, individual genetics, and underlying pathology (Shetty et al., 2023). Specific pain conditions like neuropathic pain may necessitate early initiation of medications with neuromodulatory effects (Ammar et al., 2021). These medications encompass antidepressants (tricyclics, SSRIs, SNRIs) or anticonvulsants. The European Federation of Neurological Societies (EFNS) emphasizes the need for individualized treatment approaches for cancer pain, with drug selection based on the specific pain mechanisms involved (Caylor et al., 2019). Corticosteroids, bisphosphonates, monoclonal antibodies, and topical medications like lidocaine or ketamine may also be incorporated into pain management regimens. The WHO framework acknowledges exceptions for the use of opioid analgesics in managing acute or life-threatening conditions (Megía García et al., 2020). These situations may necessitate deviation from the strict stepwise approach. The second stage of the analgesic ladder involves transitioning to more potent medications with a higher risk of side effects. This stage primarily involves weak opioid analgesics (tramadol) or low-dose strong opioids (Inanici et al., 2021). Opioid titration starts from the minimum effective dose and may be combined with first-line medications for synergistic effects. Careful consideration should be given to the potential downsides of high-dose opioids or medications with a stronger CNS impact (Inanici et al., 2021). The final stage involves treatment with high-dose strong opioids or combinations thereof (Gilbert et al., 2022). To mitigate the risk of addiction and other adverse effects associated with high-dose opioids, coadministration with opioid receptor blockers like naloxone or naltrexone may be employed in specific cases (Gilbert et al., 2022).

A study comparing intravenous paracetamol (acetaminophen) to NSAIDs and opioids for moderate to severe acute pain in the emergency department found no significant difference in pain reduction at 30 min between the groups (Qureshi et al., 2023). Additionally, a comparison of tramadol/acetaminophen fixed-dose combination (Ultracet) with the fentanyl transdermal system for gynecologic cancer pain showed similar efficacy rates (83% vs. 89%, respectively) (Sun and Ho, 2014). Tapentadol nasal spray demonstrated superior pain reduction compared to intravenous tramadol in postoperative moderate to severe pain (Shetty et al., 2023). Furthermore, in a study on chronic severe pain, tapentadol prolonged release showed statistically significant pain reduction compared to other analgesics (Bagaphou et al., 2019).

A two-decade long-term study investigated the efficacy and safety of Spinal cord stimulation (SCS) in 692 patients suffering from chronic pain. The findings revealed an initial success rate of 85%–86% 1 month post-implantation. However, this success rate waned over time, with a long-term success rate of 52%–54% observed at a mean follow-up of 10 years (ranging from 2 to 20 years). Interestingly, the study noted an improvement in success rates during the latter period (1984–1990) involving 301 patients, reaching 60%–68%. These results support the therapeutic value of SCS, particularly for neurogenic pain arising from partial deafferentation (reduced sensory input) (Lazorthes et al., 1995).

In contrast to SCS, transcranial magnetic stimulation (TMS) presents a less definitive picture for chronic pain management. A systematic review focusing on rTMS for chronic orofacial pain indicated promising results. Repetitive stimulation applied to specific brain regions, including the motor cortex, dorsolateral prefrontal cortex, and secondary somatosensory cortex, demonstrated adequate pain relief (Ferreira et al., 2019). However, another systematic review on TMS for broader chronic refractory pain yielded mixed findings. Low-frequency rTMS showed no significant effect, and overall efficacy varied depending on stimulation parameters and targeted brain areas. This review highlights the need for further research to establish optimal stimulation protocols for improved consistency and effectiveness of TMS as a pain management tool (Hamid et al., 2019).

Finally, it is noteworthy that the long-term success rates reported for SCS (52%–68%) fall short of the 70%–90% efficacy reported for strong opioids in severe pain cases (mentioned previously) (Chow and Rosenquist, 2023). This underscores the complexity of chronic pain management and underscores the potential need for a multimodal approach, potentially combining SCS or TMS with other interventions for optimal patient outcomes.

### 5.2 Non-pharmacological treatments for neuropathic pain

#### 5.2.1 Spinal cord stimulation (SCS)

Initially introduced by Melzack and Wall in 1965, the gate control theory of pain, now known as the gate control theory of pain, led to various attempts at CNS electrical stimulation for pain treatment in the fifties and sixties. Two years later, Shealy and colleagues introduced SCS. The first method used was open intrathecal electrode implantation via laminotomy (Rogers et al., 2022). In recent years, SCS has become popular as a treatment for persistent low back pain. This is due to its reduced invasiveness and reversibility compared to nerve ablation. New developments in hardware design have also led to longer device life and a simplified implantation process. Before permanent implantation, a cervical SCS trial is conducted as a less invasive treatment to allow patients to experience SCS (Beauchene et al., 2023). Scientific evidence shows better outcomes from this modality compared to other treatments for certain forms of low back pain. SCS is the most common form of neuromodulation used for chronic low back pain. The main indication for this type of stimulation is failed back surgery syndrome (Dalrymple et al., 2022). Depending on the evaluation of accessibility to the epidural space, it is often implanted in the epidural space using either a surgical or percutaneous approach. Dorsal column stimulation is the term used to refer to the stimulation of the spinal cord caused by the electrodes during SCS. SCS was once considered a last-resort treatment for persistent low back pain, but many interventional pain practitioners now attempt it early in the condition (Graham, 2022). However, due to the low costs of SCS trials relative to other interventions, its low risk/benefit ratio, and favorable outcome studies, SCS may be the best treatment for some forms of chronic low back pain such as failed back surgery syndrome. Although the mechanisms of action are attributed to Melzak and Wall’s “gate control theory,” recent research efforts have shown new probable mechanisms of action. SCS may act, at least in part, by modulating neurotransmission from the CNS (Cantero, 2023). SCS is indicated for complex regional pain syndrome (CRPS), failed back surgery syndrome (FBSS), and refractory angina pectoris. The number of implanted spinal cord stimulators has significantly increased in the last 20 years. A few other potential therapeutic effects are supported by SCS. These include pain relief, improved function, and improved quality of life (Rogers et al., 2022).

#### 5.2.2 Transcranial magnetic stimulation (TMS)

Transcranial Magnetic Stimulation (TMS) is a non-invasive, safe process that uses an electromagnetic coil to create a magnetic field. This generates transient magnetic pulses that can activate the brain cortex and pass through the skull with minimal hindrance. These pulses alter cortical excitability at the site of stimulation and as mentioned earlier, also have transynaptic effects in distant regions (Samejima et al., 2022). Repetitive TMS (rTMS) refers to the repeated application of TMS pulses. rTMS has been found to enhance motor and cognitive functions, as well as reduce depressive symptoms in various disorders including stroke, Parkinson’s disease, and major depressive disorder. It has also been reported to have pain-reducing effects in other pain-related conditions. The areas of the brain associated with pain perception include the hypothalamus, amygdala, thalamus, somatosensory cortex, insula, ACC, and prefrontal cortex (Sayenko et al., 2019). Different clinical outcomes are observed in patients with painful conditions. Acute pain conditions that follow normal healing processes usually resolve after a certain period, but in some cases, they progress to become chronic pain conditions that significantly affect patients’ quality of life. Conventional therapies such as medications, anesthetics, corticosteroid injections, and behavioral therapies may fail in these patients (Seghier, 2023). Recent advancements in neurostimulation techniques, including deep brain stimulation, motor cortex stimulation, SCS, peripheral nerve stimulation, and rTMS, have made it easier to manage chronic pain. rTMS is used in combination with other treatments to modulate aberrant brain activity and alleviate pain. The relief of pain achieved through cortical stimulation is based on changes in neuronal excitability (Siebner et al., 2022). The specific locations targeted for activity changes with rTMS, in relation to pain modulation and processing in cortical and subcortical brain structures, include the orbital frontal cortices, anterior cingulate, medial thalamus, and periaqueductal gray matter. rTMS also modulates chronic pain by activating descending inhibitory neural pathways at the dorsal horn level. It has been established that rTMS can regulate the functioning of neurons in the periaqueductal gray matter during the pain process (Corneille and Lush, 2023). The frequency of stimulation is associated with synaptic alterations; low frequencies (<1 Hz) have inhibitory effects, while high frequencies (>5 Hz) have excitatory effects. High-frequency stimulation increases cortical excitability, while low-frequency stimulation decreases it. The stimulation frequency can be adjusted based on the site of stimulation and the patient’s level of discomfort (Segil et al., 2022).

## 6 Challenges and opportunities

This is based on the rationale that the innate analgesic system also exhibits variability in activity over time. This variability is one of the reasons why patients with chronic pain demonstrate such a wide range of responses to both pharmacological and non-pharmacological treatments. Moreover, the external administration of α2-agonists, opioids, and cannabinoids can disrupt natural systems, leading to an imbalance that contributes to the development of various disease conditions (Oh et al., 2023). Long-lasting pain is caused by impairments to the somatosensory peripheral nervous system (Table 1), such as those caused by diabetes or herpes, which block sensory signals sent to the brain and spinal cord. Given that medication-induced chronic pain often diminishes quality of life and increases social and medical expenses (Gu et al., 2023), it is crucial to identify more effective drugs for chronic treatment. Among the drugs used to treat different pain syndromes, such as opioids, NSAIDs, selective COX-2 inhibitors, antiepileptics, antidepressants, anticonvulsants, and local anesthetics, there is another social and clinical problem–the opioid crisis caused by excessive use of opioids in the treatment of chronic pain (Sil et al., 2018). A critical issue regarding the use of opioids is that prolonged use can result in the development of tolerance and physical dependence (Huang et al., 2019d). The challenges associated with their clinical use and the undesirable side effects experienced by patients have prompted efforts to develop opioids with fewer adverse effects, such as those resulting from biased opioid agonism, multifunctional opioids, and allosteric modulation of opioid receptors (‏Mibielli et al., 2020). Novel analgesics have been developed using receptor targets such as adrenergic, cannabinoid, P2X3 and P2X7, NMDA, serotonin, and sigma receptors, as well as voltage-gated sodium channels, Nav1.7 and Nav1.8 (Selvy et al., 2021). Other enzymes being considered for the development of a new class of analgesics include soluble epoxide hydrolase, sepiapterin reductase, and MAGL/FAAH, among others. Chronic opioid therapy for treatment-resistant chronic pain often comes with a high risk of questionable benefit, increasing the need for additional types of pain control interventions (Matesanz et al., 2021). Chronic neuropathic pain (NP) is the most challenging clinical condition to treat. First-line therapies that modify symptoms include a range of medication approaches combined with careful non-pharmacological treatments (Xu et al., 2023). However, in cases where drug therapies are highly ineffective or not tolerated by patients (Mimenza Alvarado and Aguilar Navarro, 2016), interventional neuromodulation techniques using modern implantable technology for long-term delivery of electrical stimulation to the nervous system should be considered. Spinal cord and peripheral nerve stimulation can be used in patients with chronic refractory NP caused by lesions located elsewhere in the peripheral nervous system (Mibielli et al., 2020). Spinal cord stimulation (SCS) has been used in the treatment of chronic pain for the past 4 decades and has been approved by the Food and Drug Administration in the United States for chronic pain of the trunk and extremities. Deep brain electrical stimulation (DBS) and epidural motor cortex stimulation (MCS) are invasive surgical treatments that are highly challenging to apply in chronic NP cases of central origin (‏Lee et al., 2022). In general, DBS yields poor results in chronic NP patients and is only used in a small subset of patients who have not responded to other interventions (Xu et al., 2023). Although MCS has achieved significant pain reduction in some subgroups of NP patients, difficulties in selecting the right patient and optimizing and standardizing stimulation parameters have limited its use (Sharma et al., 2021). Repetitive TMS (rTMS) is a non-invasive neuromodulation technique used for pain treatment by stimulating the cerebral cortex through a transcranial magnetic field. It is based on the electrical currents produced by the applied magnetic field and can have immediate and long-lasting effects on cortical excitability (Gatzinsky et al., 2021).

**TABLE 1:** Provides a concise overview of the results obtained from the pain trials that are included in the analysis.

Authers	Cause of pain	Study type	Intervention assessed	Limitations	Main outcome	Ref
Gu, Jialin, et al., 2023	Chemotherapy induced peripheral neuropathy	RCT	Acupuncture plus intramuscular methylcobalamin initially then oral methylcobalamin *versus* methylcobalamin intramuscularly then orally alone	Open label. No placebo arms. Poor Jadad score	Both treatment groups showed a significant reduction in pain measured by visual analog scale (VAS). Although improvement was greater in the acupuncture group, it was not significant	Oh et al. (2023b)
Sil, Amrita, et al., 2018	Peripheral polyneuropathy	RCT	Intramuscular methylcobalamin of two different doses	No placebo arms. Small population size	There was a significant reduction in pain measured by Leeds assessment of neuropathic signs and symptoms (LANSS) and Douleur Neuropathique 4 (DN4)	Praveen et al. (2022)
Huang, Zhi-Fa, et al., 2019	Piriformis syndrome	Single armed interventiona	Intravenous mannitol plus oral B1, B2 and B12	Small study size. No placebo arms. Open label	There was a significant reduction in pain evaluated by NRS and LRS.	Alkhubaizi et al. (2022)
Mibielli, Marco Antonio Naslausky, et al., 2020	Degenerative orthopedic alteration with neural compression	RCT	Oral cytidine monophosphate disodium uridine triphosphate trisodium and hydroxocobalamin *versus* oral hydroxocobalamin alone	Short follow-up. No placebo arms	Both arms had a statistically significant reduction in pain measured by VAS, but the improvement was greater in the arm inclusive of nucleosides	Bonezzi et al. (2020)
Selvy, Marie, et al., 2021	Chemotherapy induced peripheral neuropathy	RCT	Placebo *versus* oral capsule of thiamine, riboflavin, niacin, pantothenic acid, pyridoxine, folate (B9), cyanocobalamin, biotin, choline, and inositol	This study was underpowered. Small study size	The vitamin capsule did not decrease the incidence of pain compared with placebo	Bisset et al. (2023)
Matesanz, Luis, et al., 2021	Peripheral entrapment neuropathy	Single armed interventional	Oral uridine monophosphate, folic acid, and vitamin B12	No placebo arms. Open label. Did not include severe neuropathies. A very small dose of B12 is used. Small study size	There was a significant reduction in pain assessed by PDQ score and a reduction in concomitant use of analgesics	Marotta et al. (2022)
Xu, Gang, et al., 2023	Acute ophthalmic herpetic neuralgia	RCT	Lidocaine and methylcobalamin subcutaneously locally *versus* intramuscularly	The subcutaneous locally delivered treatment showed a decrease in pain severity a year later. No placebo arms. Small study size	While the intramuscular group showed a statistically significant reduction in pain measured by NRS, this was short-lived and tapered off after 14 days	Alrizqi and Aleissa (2023)
Mimenza Alvarado, Alberto, et al., 2016	Diabetic peripheral neuropathy	Non-randomized controlled study	Oral gabapentin, thiamine, and cyanocobalamin *versus* pregabalin alone	Did not include patients with severe neuropathy. Open label. Non-randomized. No placebo arms	Both groups showed a statistically significant improvement in pain measured by VAS. Both arms experienced less interrupted sleep. There was no difference between arms	Alrashdan et al. (2019)
Mibielli, Marco Antonio Naslausky, et al., 2020	Peripheral polyneuropathy	Single armed interventional	Oral capsule containing uridine monophosphate, B12 and folic acid	Open label study design. No placebo groups	There was a significant reduction in PainDETECT questionnaire (PDQ) and a reduction in analgesia use	Gupta et al. (2018)
Lee, Chang-Woo, et al., 2022	Diabetic peripheral neuropathy	Single armed interventional	Oral L-methylfolate, methylcobalamin and pyridoxal-5-phosphate	No placebo groups. Open label. Adverse events were not reported	There was a significant reduction in pain captured by VAS and NTSS6. There was an improved quality of life with a focus on pain	Iannicelli et al. (2019)
Xu, Gang, et al., 2023	Truncal post-herpetic neuralgia	RCT	Lidocaine and methylcobalamin subcutaneously locally *versus* intramuscularly	No placebo arms	There was a more significant reduction in pain measured by NRS and improvement in quality of life in those receiving local subcutaneous injection over the intramuscular group	Alahmary (2019)
Sharma, Chetna, et al., 2021	Peripheral polyneuropathy	Post-marketing surveillance study	Oral pregabalin plus methylcobalamin	Observational study design. Short follow-up	Reduction in VAS for pain and a reduction in concomitant analgesia use	Mallari et al. (2019)

RCT, Randomized controlled trials.

For a more comprehensive evaluation, we could have used additional tools, such as the Cochrane Risk of Bias Tool for randomized controlled trials and appropriate tools for non-randomized studies.

## 7 Ethical and legal considerations

When investigating the molecular mechanisms and neurochemical pathways underlying pain, researchers must carefully consider the ethical implications of their work. Pain research often involves animal models, which raises concerns about animal welfare and the ethical treatment of research subjects (Oakes et al., 2022). Researchers must ensure that animal experiments are designed to minimize suffering and that the potential benefits of the research outweigh the risks to the animals. Additionally, pain research may involve human participants, either in clinical trials or observational studies. Researchers must obtain informed consent from participants and protect their privacy and confidentiality. They must also consider the potential risks and benefits of the research to human participants and ensure that the study design is ethically sound (Alireza et al., 2014; Coetser, 2023).

The legal landscape surrounding pain research is complex and varies across different districts. Researchers must ensure that their work complies with all relevant laws and regulations, including those related to the use of animals in research, the protection of human research participants, and the handling of sensitive medical data (Alireza et al., 2014; Adhikari et al., 2024). In some cases, pain research may involve the development of new drugs or medical devices, which are subject to stringent regulatory approval processes. Researchers must work closely with regulatory agencies to ensure that their work meets all legal requirements and that any new treatments or interventions are safe and effective (Coetser, 2023; Adhikari et al., 2024).

Overall, researchers in the field of pain must navigate a complex web of ethical and legal considerations to ensure that their work is conducted in a responsible and ethical manner while also advancing our understanding of the molecular mechanisms and neurochemical pathways underlying pain.

## 8 Conclusion

The management of NP in patients remains challenging, despite significant advances in our understanding of the condition. Patient satisfaction with treatment is generally low. NP is caused by central sensitization, the activation of immune cells, and the release of proinflammatory mediators following damage to the peripheral nervous system. Due to the multifactorial nature of NP and its underlying mechanisms, clinicians must address various levels of mechanisms and consider any comorbid conditions in order to provide better pain control. This mechanism-based approach has the potential to significantly improve patients’ quality of life. Currently, opioids, non-steroidal anti-inflammatory drugs, and antidepressants are the mainstays of pain management strategies, although opioids are associated with physical dependence and tolerance issues. There are several options available for pain management, such as neurostimulation techniques like rTMS and SCS. The development of new pharmacotherapeutics, along with meticulous clinical trials and a better understanding of the contribution and mechanisms of neuroplasticity, will undoubtedly advance the clinical treatment and prevention of NP.

## Figures and Tables

**Table:** 

**ACC**	Anterior Cingulate Cortex
**AMPA**	α-amino-3-hydroxy-5-methyl-4-isoxazolepropionic acid
**ASICs**	Acid-Sensing Ion Channels
**BLA**	Basolateral Amygdala
**CeA**	Central Amygdala
**CGRP**	Calcitonin Gene-Related Peptide
**CNS**	Central Nervous System
**CRF**	Corticotropin-Releasing Factor
**DOR**	Delta Opioid Receptor
**DRG**	Dorsal Root Ganglia
**GABA**	Gamma-Aminobutyric Acid
**ITC**	Intercalated Cells
**KOR**	Kappa Opioid Receptor
**MAGL/FAAH**	Monoacylglycerol Lipase/Fatty Acid Amide Hydrolase
**mGlu**	Metabotropic Glutamate
**MOR**	Mu Opioid Receptor
**NAAG**	N-acetylaspartylglutamate
**NK1**	Neurokinin 1
**NMDA**	N-methyl-D-aspartate
**NPS**	Neuropeptide S
**OT-R**	Oxytocin Receptor
**P2X3**	P2X Purinoceptor 3
**P2X7**	P2X Purinoceptor 7
**PNS**	Peripheral Nervous System
**pNP**	Peripherally Induced Neuropathic Pain
**SOM**	Somatostatin
**TRPM8**	Transient Receptor Potential Melastatin 8
**TRPV1**	Transient Receptor Potential Vanilloid 1
**TRPV2**	Transient Receptor Potential Vanilloid 2
**V1aR**	Vasopressin 1a Receptor
